# Effects of Dietary Chlorogenic Acid Supplementation on Growth Performance, Meat Quality, and Muscle Flavor Substances in Finishing Pigs

**DOI:** 10.3390/foods12163047

**Published:** 2023-08-14

**Authors:** Kunhong Xie, Yaxin Sun, Lili Deng, Bing Yu, Yuheng Luo, Zhiqing Huang, Xiangbing Mao, Jie Yu, Ping Zheng, Hui Yan, Yan Li, Hua Li, Jun He

**Affiliations:** 1 Institute of Animal Nutrition, Sichuan Agricultural University, Chengdu 625014, China; xiekunhong20130234@163.com (K.X.); syaxin57866@163.com (Y.S.); ybingtian@163.com (B.Y.); luoluo212@126.com (Y.L.); zqhuang@sicau.edu.cn (Z.H.); xiangbingm@hotmail.com (X.M.); yujie@sicau.edu.cn (J.Y.); zpind05@163.com (P.Z.); yan.hui@sicau.edu.cn (H.Y.); m18782044216@163.com (Y.L.); spbee1757@163.com (H.L.); 2 Key Laboratory for Animal Disease-Resistance Nutrition of China Ministry of Education, Chengdu 625014, China; 3 College of Veterinary Medicine, Sichuan Agricultural University, Chengdu 625014, China; ly18280157033@outlook.com

**Keywords:** chlorogenic acid, carcass traits, meat quality, flavor, antioxidant properties

## Abstract

With the prohibition of antibiotics in feed, certain phytocompounds have been widely studied as feed additives. Chlorogenic acid (CGA), a natural polyphenol found in plants, possesses anti-inflammatory, antioxidant, and metabolic regulatory features. The objective of this study was to investigate the effects of dietary chlorogenic acid supplementation on growth performance and carcass traits, as well as meat quality, nutrient value and flavor substances of Duroc × Landrace × Yorkshire (DLY) pigs. Forty healthy DLY pigs (initial body weight (BW): 26.69 ± 0.37) were allotted to four treatment groups and were fed with the control diet, which was supplemented with 25 mg kg^−1^, 50 mg kg^−1^, and 100 mg kg^−1^ CGA, respectively. The trial lasted 100 days. The results suggested that dietary CGA supplementation had no effect (*p* < 0.05) on the average daily gain (ADG) and feed conversion ratio (FC). Herein, it was found that 50 mg kg^−1^ CGA-containing diet not only increased the dressing percentage and perirenal fat, but also reduced the rate of muscular pH decline (*p* < 0.05). In the longissimus thoracis (LT) muscle, the myofiber-type-related genes such as the MyHC IIa and MyHC IIX mRNA levels were increased by 100 mg kg^−1^ CGA. The results also indicated that the 100 mg kg^−1^ CGA-containing diet increased the content of crude fat, glycogen, total amino acids, and flavor amino acids, but decreased the inosine and hypoxanthine concentration in LT (*p* < 0.05). Meanwhile, the lipogenic gene *ACC1* mRNA level was elevated by 50 mg kg^−1^ CGA. Instead, 100 mg kg^−1^ CGA downregulated the expression level of NT5C2, an enzyme responsible for inosine-5′-monophosphate (IMP) degradation. Additionally, 100 mg kg^−1^ CGA decreased the malondialdehyde (MDA) content, but increased the glutathione peroxidase (GSH-Px) content as well as antioxidant gene (*HO-1*, *NQO-1*, *NRF2*) mRNA levels in LT muscle. These findings showed that dietary CGA could partly improve carcass traits and muscle flavor without negatively affecting growth performance, and the underlying mechanism may be due to the antioxidant properties induced by CGA.

## 1. Introduction

With the ongoing intensification of swine production, pigs are susceptible to oxidative stress due to a variety of stressors, including weaning, vaccination, nutrition/metabolism, disease, transportation, and environmental and social stress [1]. There is accumulating evidence that oxidative stress adversely affected growth performance [2], decreased antioxidant properties of tissues [3], and deteriorated meat quality [4] and flavor [5] of pigs. Recently, there has been increasing consumer demand for higher quality meat. In this context, to ensure continued development of the pig farming industry, it is necessary to take measures to prevent the deterioration of pork quality caused by oxidative stress, especially through dietary strategies. Previous studies showed that dietary supplementation with certain phytocompounds, such as polysaccharides and polyphenols, is an effective approach to address this issue [6,7].

Chlorogenic acid (CGA) is known as a natural water-soluble polyphenol formed by esterification of trans-cinnamic and quinic acids, which is found in various foods and herbs, including coffee beans, artichoke, eucommia, grapes, kiwi fruit, honeysuckle, wormwood, and tea [8]. Numerous studies have showed that it has a variety of bioactive properties, including being anticancer [9], anti-diabetic [10], antihypertensive [11], anti-inflammatory, and anti-oxidant [12]. Hence, CGA is considered to have a broad application prospect in providing a potential benefit for the animals’ overall productivity and meat product quality. It is worth noting that foremost amongst the biological properties of CGA is antioxidant activity, and chlorogenic acid is superior to vitamin E in scavenging free radicals in vitro [13,14]. From an application standpoint, CGA may work as an anti-stress feed additive to alleviate the deterioration of pork quality caused by oxidized corn oil [15]. In a recent study by Wang et al., providing dietary CGA to pigs could reduce the lightness of muscle by inducing a shift in muscle fiber type to slow-twitch fibers [16]. Additionally, an intriguing study has confirmed that the addition of chlorogenic acid can inhibit lipid oxidation, the formation of protein carbonyl group, and the loss of tryptophan, which significantly improves the physical properties of pork [17].

Previous studies have indicated that CGA is associated with many aspects of meat quality, such as color, drip loss, water-holding capacity, ultimate pH (pHu), etc. [18,19,20]. Comparatively, the literature contains much less information on the effects of chlorogenic acid on meat flavor. It is commonly reported that pork flavor is affected by taste-active components such as inosine-5′-monophosphate (IMP) and free amino acids responsible for flavor [21]. However, excessive metabolism of IMP will accumulate inosine and hypoxanthine, leading to a bitter taste in meat [22]. Additionally, the Maillard reaction between amino acids and reducing sugars will yield hundreds of volatile flavor compounds that contain an N, O, or S atom in their ring structure [21]. In the literature, there are some studies on the application of CGA in swine nutrition [15,16]. So far, information about the effects of chlorogenic acid on meat’s nutritional value and flavor substances in finishing pigs is limiting, and the underlying mechanism remains to be established. Hence, the aim of the present study was to assess the effectiveness of dietary CGA supplementation on growth performance, meat quality parameters and flavor substances. The mechanisms of chlorogenic acid on meat quality were also investigated in this study.

## 2. Materials and Methods

### 2.1. Ethics Statement

This study was submitted and approved by the Committee on Animal Care Advisory of Sichuan Agricultural University (authorization number SICAU-2021-007). All experiment procedures were performed following the Guidelines for the Care and Use of Laboratory Animals.

### 2.2. Animals and Diets

At an experimental farm of Sichuan Agricultural University in Yaan city, a total of 40 healthy three ways cross (Duroc × Landrace × Yorkshire) pigs (average 26.69 ± 0.37 kg) were randomly allocated to four treatment groups (control group, 25 mg kg^−1^ CGA group, 50 mg kg^−1^ CGA group, 100 mg kg^−1^ CGA group) with 5 replicates in each group and 2 pigs in each replicate. The control diets adapted a corn–soybean meal basal diet and were formulated to meet the recommendations of the National Research Council [23] and the different breeding phases of pigs (Table 1). When formulating the feed for the treatment group, the 25, 50, or 100 mg of CGA powder was added at the expense of equal amount of basal feed, respectively. The CGA was purchased from Guilin Fengpeng Biotechnology Co., Ltd. (Guilin, China), and the main active ingredients included 50% chlorogenic acid. The diets were added in the troughs three times a day (8:30, 14:30 and 20:30 h) to make sure they had fresh feed and drinking water ad libitum. All pigs were housed in the barn where the average temperature and average humidity were 23.0 ± 2.54 °C and 75.5 ± 0.12%, respectively. The feed intake and weight were recorded at the 0 d, 29 d, 53 d, 80 d, and 100 d of the experimental period to calculate the average daily feed intake (ADFI), average daily gain (ADG), and feed conversion ratio (FCR) for each phase.

### 2.3. Sample Collection

From day 96 to day 100 of the feeding trial, the fecal samples were taken via rectal massage, pooled and used for determination of nutrient digestibility. Before drying in an oven at 60 °C, fecal samples were treated with 10% hydrochloric acid for nitrogen fixation. The dried samples were passed through a 0.25 mm filter for chemical assay.

At the end of the experiment, one pig was randomly selected from each pen (5 pens in each group and 2 pigs in each pen) and slaughtered after fasting for 12 h. Before slaughter, about 5 mL blood samples were taken from anterior vena cava and centrifuged (3000× *g* for 20 min at 4 °C) to collect serum. The serum samples were stored at −20 °C for the following determination. At the slaughterhouse, each pig was electrically stunned (300 V for 3 s) and exsanguinated. After the carcass was scalded, dehaired, peeled, and eviscerated, it was split down from the midline. Thereafter, the left side of carcass was used for measurements of meat quality parameters. Approximately 600 g of the left longissimus thoracis (LT) muscle was sampled. Some LT subsamples (100 g each) were immediately stored at 4 °C for the determination of shear force, water-holding capacity, pH, and meat color at 24 h post mortem. Additional LT samples (20 g) were taken and fixed in 4% paraformaldehyde for hematoxylin and eosin staining. The remaining LT subsamples (180 g) were sliced, individually vacuum-packed, and stored at −80 °C for the analysis of chemical composition, amino acid composition, antioxidant capacity and real-time PCR (RT-PCR).

### 2.4. Chemical Analysis

The nutrient composition of diets and fecal samples was determined based on the international standard methods described by the AOAC International [24], including dry matter (930.15; AOAC), crude protein (930.15; AOAC), crude fat (920.39; AOAC), crude ash (942.05; AOAC), crude fibre (978.10; AOAC) and Ca (968.08; AOAC). The gross energy was analyzed by using a 6400 automatic adiabatic oxygen bomb calorimeter (Parr Instrument, Moline, IL, USA). For digestion test, the endogenous indicator acid-insoluble ash (AIA) standard method (GB/T 23742–2009) was used in the present study [25].

### 2.5. Serum Biochemical Parameters

The concentrations of serum glucose, triglyceride, total cholesterol, high-density lipoprotein cholesterol, and low-density lipoprotein cholesterol were assayed using GLU (Cat. No. F006-1–1), TG (Cat. No. A110-1–1), T-CHO (Cat. No. A111-1–1), HDL-C (Cat. No. A112-1–1), and LDL-C (Cat. No. A113-1–1) commercial kits, respectively. Each test was run in duplicate using an automatic analyzer (Olympus, Shanghai, China).

### 2.6. Carcass Traits

After slaughter, the live weight and hot carcass weight (HCW) were recorded within 5 min so that the dressing percentage could be calculated by dividing the carcass weight by the live weight. Within 30 min after slaughter, the carcass was split in half along the midline, and all carcass traits measurements (the length of carcass, average backfat depth, loin muscle area, and carcass lean percentage, etc.) were finished. Briefly, carcass length was defined as from the leading edge of the first rib to the pelvic joint; the backfat depth of the first, 10th and last ribs of the left carcass was measured to calculate the average backfat depth; and a loin muscle (LM) chop (2.5 cm thick) was taken from 10th rib to the 13th rib to determinate the area of loin muscle. The contour of loin muscle area at the 10th rib of the carcass was measured with a planimeter by tracing onto transparent paper. The carcass lean percentage was measured by using the following formula: Lean (%) = (fat-free carcass weight/the carcass weight) × 100. The weights of skin and perirenal fat at slaughter were also recorded.

### 2.7. Muscle Physical Traits

Within 24 h post mortem, the fresh Longissimus thoracis muscle samples were taken from the 10th and 16th ribs. After removing the connective tissue and surface fat, the longissimus thoracis muscle was cut into small pieces for determination of the pH, color, cooking loss, drip loss, and shear force in the following order.

Briefly, the measurements of pH_45min_ and pH_24h_ post mortem were performed in the longissimus thoracis muscle using an SFK-Technology pH meter equipped with a coupled penetration probe and thermometer (pH-STAT, SFK-Technology, Herlev, Denmark). Prior to measurement, the pH meter was calibrated in standard phosphate buffer at pH 7.00 and 4.01 and adjusted to the actual temperature of the sample measurements according to the instrument instructions. After exposing the freshly cut surface to 15 min of blooming, the meat color parameters of the longissimus thoracis muscle, including L* (Lightness), a* (Redness) and b* (Yellowness), were measured by CR-300 Minolta Chroma Meter (Minolta Co., Ltd., Osaka, Japan) equipped with an 8 mm aperture, D65 illuminant, and 10° permanent observer.

The water-holding capacity of the longissimus thoracis muscle was evaluated by drip loss. In short, the fresh LT samples were trimmed into a 3 cm thick chop (about 30 g), weighed on an analytical scale (the initial cube weight) and hung up vertically by a fishhook in plastic containers at 4 °C for 24 h. After removal from the plastic containers, the samples were wiped dry and reweighed (the drip cube weight). Drip loss was calculated as the difference (%) from the initial cube weight after 24 h. For cooking loss determination, the approximately 100 g fresh longissimus thoracis muscle samples were trimmed into 25 mm thick slices and weighed (W1). The same samples were cooked together in a thermostatic bath at 75 °C until the central internal temperature reached 70 °C. After this period of time elapsed, the samples were cooled at 4 °C for 30 min, drained, and weighed again (W2). The cooking loss value is calculated as follows: cooking loss (%) = (W1 − W2)/W1 × 100.

For the shear force determination, approximately 100 g of samples were taken from the longissimus thoracis muscle. For each muscle sample, 6 duplicate slices (1.27 cm diameter) were cut with a cylindrical core drill in a direction parallel to the muscle fibers. Care was taken to avoid areas where fat and connective tissue were visible during sampling. These round samples were placed in a boiling water bath until the central internal temperature reached 72 °C. After heat treatment, the samples were cooled at 4 °C for 24 h and sheared perpendicularly to the longitudinal orientation of the muscle fibers using a texture analyzer (Stable Micro System, Godalming, UK) equipped with 60° angle and a knife blade with 1.016 mm thickness. The test was performed in “compression” mode with a crosshead speed of 200 mm/min and preload force of 2 N. Shear force data were recorded as the average of six force recordings of each muscle sample.

### 2.8. Muscle Histological Analysis

The histological analysis was performed on the fibers of the longissimus thoracis muscle by hematoxylin staining and eosin staining. Briefly, muscle cores samples (5 mm^3^) were excised from the 3 × 3 × 3 cm cubes along the muscle fibers and immobilized with paraformaldehyde for 48 h. After being embedded in paraffin, these samples were cut into 4 μm sections using the CM1850 freezer (Leica Microsystems Inc., Buffalo Grove, IL, USA) in a direction perpendicular to the muscle fibers. The sections were dewaxed with xylene and rehydrated with ethanol, then stained with alcoholic eosin and methylene blue staining, and examined under a microscope. The light microscopy (Olympus, Tokyo, Japan) was used to observe the fiber sections at 100× magnification. In each section, 3 areas were randomly selected to be photographed for further analysis. The mean diameter and fiber number of longissimus thoracis muscle fibers were determined by using Image-Pro software (Media Cybernetics Inc., Rockville, MD, USA). Moreover, the total fiber count of the longissimus thoracis muscle was calculated from the fiber count and muscle cross-sectional area of the selected area.

### 2.9. Muscle Chemical Analysis

The longissimus thoracis muscle (approximately 50 g) was cut into small pieces for determination of the crude protein, crude fat, glycogen content, and flavor nucleotide content in the following order. In short, the crude protein content of samples was measured according to the procedure of AOAC International (930.15; AOAC, 2007 method) [24]. For the measurement of crude fat, the lipids were extracted from muscle samples in line with the standard method of Soxhlet extraction [26]. For the measurement of glycogen content, the samples were treated according to the detailed protocol of the glycogen assay commercial Kit (Cat. No. A043-1-1). An UV–VIS benchtop spectrometer (MAPADA, Shanghai, China) was used to determine absorbance values at 620 nm. Approximately 100 mg of longissimus thoracis samples were combined with 900 µL of precooled 0.9% saline to determine the flavor nucleotide content. The mixture was homogenized using micro-tube pestles at 4 °C and then centrifuged (5000× *g*, 10 min) to obtain the supernatant. The contents of flavor nucleotide included inosine-5′-monophosphate, hypoxanthine, and inosine in the supernatant were determined in accordance with the manufacturer’s instructions of commercial kits (Shanghai Mei Lian, Shanghai, China).

### 2.10. Amino Acid Composition Analysis

The longissimus thoracis muscle samples were trimmed into 25 mm thick slices (approximately 5 g) and freeze-dried in a freeze dryer set at −50 °C for 48 h. The dried samples were then pulverized and put through 0.45 pm filters for amino acid composition analysis. After the post-column derivatization of ninhydrin, the amino acids composition of the samples was evaluated by using an L-8900 automatic amino acid analyzer (Hitachi, Tokyo, Japan) with the following procedures. Briefly, weigh 100 mg dry LT sample into glass bottle, then add 10 mL 6 mol HCl. Nitrogen was filled into the bottle and hydrolyzed with a temperature of 110 °C for 23 h. After hydrolysis, all the hydrolysate products were transferred to a 50 mL volumetric bottle and diluted with ultra-pure water for calibration. Subsequently, the solution was filtered into an automated sampling bottle through 0.45 μm membrane filters, followed by amino acid analysis.

### 2.11. Antioxidant Capacity Analysis

The activities of malondialdehyde, catalase, total antioxidant capacity, glutathione peroxide, and total superoxide dismutase in serum were evaluated using MDA (Cat. No. A003-1–2), CAT (Cat. No. A007-1–1), T-AOC (Cat. No. A015-1–2), GSH-Px (Cat. No. A005-1–2), and T-SOD (Cat. No. A001-1–1) commercial kits obtained from Nanjing Jiancheng Bioengineering Institute (Nanjing, Jiangsu, China), respectively.

Meanwhile, each LT sample (about 0.1 g) was combined with 900 µL of precooled 0.9% saline. The mixture was homogenized using micro-tube pestles at 4 °C and then centrifuged (5000× *g*, 20 min) to obtain the supernatant for the determination of malondialdehyde and enzyme activity at appropriate dilutions. The concentrations of MDA, GSH-Px, CAT, T-AOC, T-SOD, and total protein in LT were measured in triplicate on a spectrophotometer with the above Nanjing Jiancheng commercial kits (Nanjing, Jiangsu, China).

### 2.12. RNA Extraction and Real-Time Quantitative PCR

For RNA extraction, RNAiso Plus reagent (TaKaRa, Dalian, China) was used to extract the total RNA from LT. After analyzing integrity using agarose gel electrophoresis, RNA purity and concentration were quickly determined on a spectrophotometer (NanoDrop-ND2000, ThermoFisher Scientific, Inc., Waltham, MA, USA). Subsequently, about 1.0 ug RNA sample was reverse-transcribed into complementary DNA (cDNA) for RT-PCR. As shown in Table 2, the primers designed with Primer 5 software were used to amplify target gene fragments. The target genes evaluated in the LT muscle were ACC1, acetyl coA carboxylase 1; FASN, fatty acid synthase; PEPCK, phosphoenolpyruvate carboxykinase; NT5C2, 5′-nucleotidase, cytosolic II; AMPD1, adenosine monophosphate deaminase 1; HPRT1, hypoxanthine phosphoribosyl transferase 1; NQO-1, NAD(P)H quinone dehydrogenase; NRF2, NFE2-like bZIP transcription factor 2; HO-1, heme oxygenase 1; KEAP1, kelch-like ECH-associated protein 1; and myosin heavy chain isoform genes (MyHC I, MyHC IIa, MyHC IIx, and MyHC IIb). The SYBR green kit (TaKaRa, Beijing, China) was used to conduct real-time quantitative PCR reaction by a Bio-Rad iQ6 instrument (Bio-Rad, Hercules, CA, USA). The PCR cycling conditions were as follows: 40 cycles at 95 °C for 5 s and 60 °C for 40 s. The relative gene expression normalized to beta actin (β-actin) mRNA was calculated by the 2-ΔΔCt method [27].

### 2.13. Statistical Analysis

The statistical analyses of all the data were performed using the Statistical Analysis System (version 9.4; SAS Inst. Inc., Cary, NY, USA). For growth performance and nutrient digestibility, each pen was used as the experimental unit, whereas each pig served as the experimental unit for serum biochemical indexes, carcass traits, meat quality, meat composition, amino acid profile, antioxidant capacity and RT-PCR analysis. The data for growth performance and nutrient digestibility were analyzed using mixed model with the dietary treatment as fixed effect and pen as a random effect, using the following statistical model: Y = μ + αi + νj + εij, where Y is the parameter to be tested, μ is the mean, αi is the effect of the diet (i = 1, 2, 3, 4), νj is the random effect of the pen and εij is the error term. Other data were analyzed using the Statistical Analysis System based on the General Linear Model. Prior to variance analysis, Shapiro–Wilk statistical procedures were used to judge the normality of the data. One-way ANOVA was used to evaluate the effects of the dietary treatment and the differences between means were assessed using Duncan’s multiple comparison test. The experimental data were expressed as the mean ± standard error (SEM). A tendency was considered 0.05 < *p* < 0.10, and *p* < 0.05 was considered a significant difference.

## 3. Results

### 3.1. Growth Performance and Nutrient Digestibility

Dietary CGA had no significant effect on ADG and FCR in each trial period (*p* > 0.05) (Table 3). Compared to the control group, 100 mg kg^−1^ CGA-supplemented diet markedly increased the body weight at 100 d. As for nutrient digestibility, the digestibility of dry matter in the 25 mg kg^−1^ CGA group were markedly decreased (*p* < 0.05) compared to the control group (Table 4).

### 3.2. Serum Biochemical Indexes

The serum biochemical parameters of the finishing pigs fed with diets containing different doses of CGA are displayed in Table 5. More generally, dietary CGA supplementation had no effect (*p* > 0.05) on the concentrations of LDL-C, HDL-C, GLU, T-CHO, or TG in serum.

### 3.3. Carcass Traits and Muscle Physical Traits

The effectiveness of dietary CGA supplementation on the carcass traits are reported in Table 6. Compared with the control group, 50 mg kg^−1^ CGA supplementation markedly increased (*p* < 0.05) perirenal fat and dressing percentage. The carcass length, carcass weight, eye muscle area, backfat depth, and carcass lean percentage were not different among groups (*p* > 0.05).

Regarding muscle physical traits, Table 7 showed that 50 mg kg^−1^ CGA increased the PH_24h_ value of the longissimus thoracis muscle in finishing pigs (*p* < 0.05). However, no differences were observed for pork quality descriptors, including the redness (a*), lightness (L*), and yellowness (b*) values, pH_45min_ value, cooking loss, drip loss, and shear force among the four groups.

**Figure 1:**
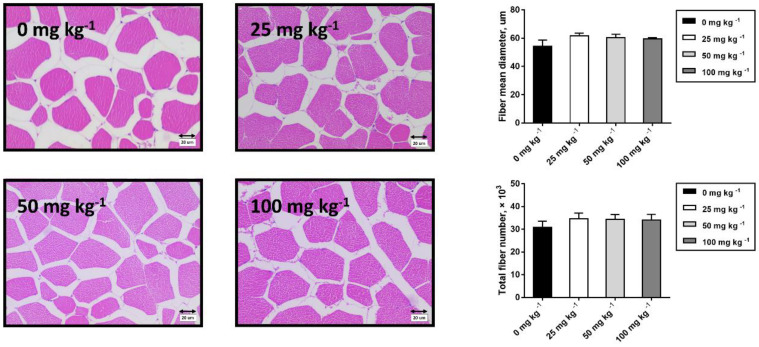
Histological analysis of *longissimus thoracis* muscle in finishing pigs fed the diets with various levels of dietary CGA. Left: Representative hematoxylin-eosin micrographs of myofiber cross sections of *longissimus thoracis* (magnification, ×400, bar = 10 μm). Right: Quantitative analysis of fiber diameter and total fiber number in *longissimus dorsi*. Data are expressed as means ± SEM (*n* = 5).

**Figure 2:**
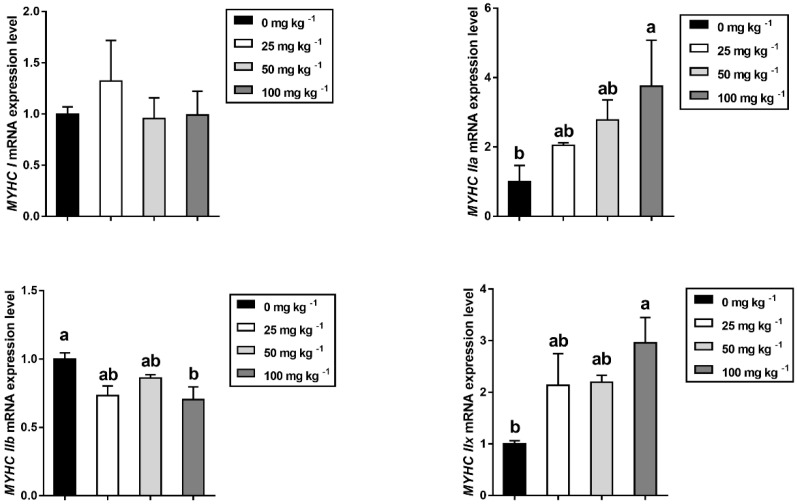
MyHC expression levels in *longissimus thoracis* muscle of finishing pigs fed the diets with various levels of dietary CGA (*n* = 5). Within a panel, bars labeled with different superscript letters significantly different at *p* < 0.05. *MyHC I*, Myosin heavy chain I; *MyHC IIa*, Myosin heavy chain IIa; *MyHC IIb*, Myosin heavy chain IIb; *MyHC IIx*, Myosin heavy chain IIx.

Muscle fiber characteristics are closely related to pork quality. Figure 1 showed the muscle fiber number and diameter in the longissimus thoracis. There was no significant difference in muscle fiber number and diameter among all groups (*p* > 0.05). As for the muscle fiber-type-related genes, Figure 2 shows that 100 mg kg^−1^ CGA supplementation markedly increased the mRNA abundance of MyHC IIa and MyHC IIx transcripts (*p* < 0.05), but reduced the mRNA abundance of MyHC IIb transcripts in LT compared to the control group.

### 3.4. Longissimus Thoracis Muscle Chemical Composition

The chemical composition of longissimus thoracis is shown in Table 8. The supplement of 50 mg kg^−1^ CGA in the feed resulted in a significant increased (*p* < 0.05) glycogen content compared to pork from pigs fed the control diet. Also, muscle crude fat content significantly increased by the 100 mg kg^−1^ dietary CGA treatment (*p* < 0.05). However, the dietary CGA inclusion had no effects on muscle crude protein and IMP content (*p* > 0.05). Additionally, dietary supplementation with 100 mg kg^−1^ CGA decreased (*p* < 0.05) the content of hypoxanthine and inosine.

### 3.5. Relative mRNA Expression of Glucose, Lipid, and Flavor Nucleotide Metabolism-Related Genes in Longissimus Thoracis

**Figure 3:**
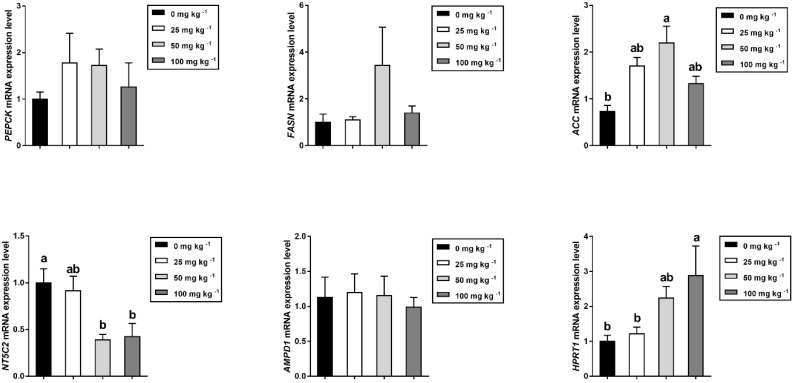
Effects of dietary CGA supplementation on relative mRNA expression of glucose, lipid, and flavor nucleotide metabolism-related genes in *longissimus thoracis* of finishing pigs (*n* = 5). Within a panel, bars labeled with different superscript letters significantly different at *p* < 0.05. *PEPCK*, Phosphoenolpyruvate carboxykinase; *FASN*, Fatty acid synthase, *ACC1*, Acetyl CoA carboxylase 1; *NT5C2*, 5′-nucleotidase, cytosolic II; *AMPD1*, Adenosine monophosphate deaminase 1; *HPRT1*, Hypoxanthine phosphoribosyltransferase 1.

Relative mRNA expression levels for metabolism-related genes in longissimus thoracis were presented in Figure 3. As for the fatty metabolism, dietary supplementation with 50 mg kg^−1^ CGA could markedly upregulate the mRNA abundance of acetyl CoA carboxylase 1 (ACC1) transcripts (*p* < 0.05) compared to the control group, but had no effects on those for FASN (fatty acid synthase). Additionally, the dietary CGA treatment affected the muscle flavor nucleotide metabolism, with a higher mRNA abundance of HPRT1 (hypoxanthine phosphoribosyl transferase 1) transcripts and a lower mRNA abundance of NT5C2 (5′-nucleotidase cytosolic II) transcripts (*p* < 0.05). There was no effect (*p* > 0.05) on mRNA abundance of AMPD1 (adenosine monophosphate deaminase 1) in this study.

### 3.6. Amino Acid Composition of Longissimus Thoracis Muscle

Amino acid composition is an important parameter to evaluate the nutrient value and flavor of meat. As shown in Table 9, the 100 mg kg^−1^ dietary CGA treatment increased (*p* < 0.05) the proportion of total amino acids (TAA) and flavored amino acids (FAA) in longissimus thoracis compared to the control treatment. Specifically, the 50 mg kg^−1^ CGA group had the highest histidine (His) proportion, but there was no difference with those of the 25 mg kg^−1^ CGA group and 100 mg kg^−1^ CGA group (*p* > 0.05).

### 3.7. Antioxidant Properties of Finishing Pigs

Antioxidant properties of serum and longissimus thoracis in pigs were shown in Table 10. The content of CAT, GSH-Px, and T-SOD in serum increased (*p* < 0.05). Regarding the antioxidant properties of muscle, the 100 mg kg^−1^ dietary CGA supplementation increased (*p* < 0.05) the GSH-Px content, but reduced the MDA content in LT (*p* < 0.05). Additionally, the content of MDA in the 50 mg kg^−1^ CGA-treated group was also decreased (*p* < 0.05). However, dietary CGA inclusion did not affect the enzyme activities of T-AOC, CAT, or T-SOD.

**Figure 4:**
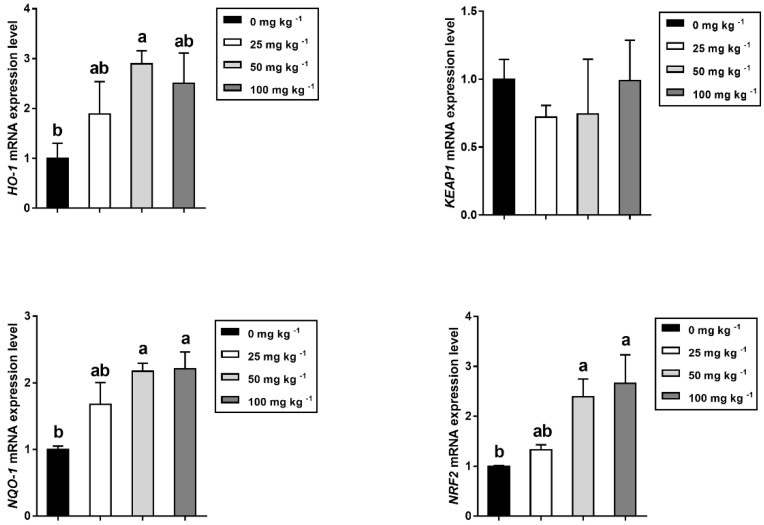
Effects of dietary CGA supplementation on relative mRNA expression of antioxidant-related genes in *longissimus thoracis* of finishing pigs (*n* = 5). Within a panel, bars labeled with different superscript letters significantly different at *p* < 0.05. *HO-1*, Heme oxygenase 1; *NQO-1*, NAD(P)H quinone dehydrogenase 1; *KEAP1*, Kelch-like ECH-associated protein 1; *NRF2*, NFE2-like bZIP transcription factor 2.

The expression levels of antioxidant-related genes in the longissimus thoracis were displayed in Figure 4. The 50 mg kg^−1^ dietary CGA supplementation markedly increased the expression of HO-1, NQO-1, and NRF2 compared to the control group (*p* < 0.05), but had no effects on those for KEAP1 in the longissimus dorsi. When the CGA supplementation dose elevated to 100 mg kg^−1^, NQO-1 and NRF2 mRNA expression levels were also increased (*p* < 0.05).

## 4. Discussion

CGA, an ester formed between quinic and caffeic acids, has been extensively studied since it possesses multiple health-promoting biological and pharmacological properties, of which antioxidative ability [13,28], muscle fiber-type conversion function [16], and potential role in glucose and lipid metabolism [29,30], as well as amino acid metabolism [31], may be pertinent to improving pork quality, nutritional value, and flavor. These observations provided impetus to investigate whether CGA improves the muscle quality, nutritional value, and flavor substances, thereby contributing to establishing the underlying mechanism. However, to the best of our knowledge, data are still lacking on this aspect in finishing pigs.

In recent years, a large number of in vivo and in vitro studies have demonstrated that CGA exerts a potential anti-obesity effect. In vivo, CGA seems to inhibit activities of G6PC (glucose-6-phosphatase) and SLC37A4 (glucose-6-phosphate translocase 1), thus controlling glucose absorption and glucose release in the small intestine [32,33]. In addition, previous studies indicated that inhibitory effects of CGA on α-amylase isozymes [34] and Lipase [35] of porcine pancreas. However, several observations have been difficult to reconcile with the assumption that the adverse influence of dietary CGA on bioavailability of nutrients might impair growth performance in pigs. Chen et al. observed that CGA inclusion diet (1000 mg/kg) in weaned pigs increased feed conversion ratio, whereas no significant effect was noted at low dietary CGA levels (250, 500 mg/kg) [36]. During the late gestation period, maternal dietary CGA (300 mg/kg) supplementation significantly increased the birth weight of neonatal pigs [37]. Another study found that the inclusion of 0.08% dietary *Eucommia ulmoides* oliver leaf polyphenol extract (containing 33.70% CGA) diet increased final body weight and average daily gain and decreased the feed intake to body gain ratio (F/G) in finishing pigs [38]. The results of this study revealed that CGA-supplemented diet did not result in differences in the main performance parameters. Along similar lines, Wang et al. (2022) have established that dietary CGA supplementation (0.02%, 0.04%, and 0.08%) derived from *Lonicera macranthoides* Hand–Mazz had no significant influence on the average daily gain and the F/G in finishing pigs [31]. Another intriguing finding in this study is that the digestibility of dry matter in the 100 mg kg^−1^ CGA group was markedly decreased (*p* < 0.05) compared to the control group. It appears, therefore, that CGA has no adverse effects on growth performance. As for the different effectiveness of dietary CGA on growth performance of pigs, one reasonable explanation might be due to the different sources or purity of CGA, as well as differences in feed types, experimental conditions, and stages of pigs.

In the present study, carcass traits, such as the carcass weight, carcass length, backfat depth, eye muscle area, and carcass lean percentage, were not affected, but the perirenal fat was markedly increased by dietary 50 mg kg^−1^ CGA supplementation. Here, it is puzzling that the effect of chlorogenic acid on increasing perirenal fat is not consistent with its anti-obesity ability [10], and the possible reasons behind this need to be further studied. In addition, 50 mg kg^−1^ CGA significantly increased dressing percentage, which is consistent with the findings of a previous study in finishing pigs [39]. For pig producers, a high dressing percentage not only increases the carcass weight available for sale, but also affects the willingness of slaughtering companies to continue buying. This study provides the first evidence that CGA-supplemented diet can increase the dressing percentage of finishing pigs, which is especially important in light of the potential for future large-scale application. In recent years, meat quality has gradually become an important objective of animal breeding. As a complex trait, meat quality is comprehensively assessed by a series of intrinsic characteristics, i.e., pH, meat color, drip loss, intramuscular fat, and shear force indicators [40]. The reason why the ultimate muscle pH can be one of the most important factors for meat quality is that the rapid pH fall in early post mortem causes more drips to be discharged from muscle fiber bundles, which indirectly affects the water-holding capacity and meat color of pork [41,42]. In this study, results indicated that 50 mg kg^−1^ dietary CGA supplementation significantly increases the PH_24h_. Additionally, the muscle drip loss value was generally lower in the dietary CGA treatment groups compared to the control group. These data agree well with previous studies that have shown that CGA reduces lactic acid production by inhibiting sarcoplasmic reticulum Ca^2+^-ATPase (SERCA) oxidation, thereby elevating the ultimate pH value of pork [15,38]. Considering that muscle fibers account for 70–90% of muscle volume, its characteristics, especially the fiber type composition and proportion in pork, are closely related to meat quality trait indicators, such as WHC, meat color, and tenderness. Based on the MyHC isoforms, muscle fibers can be divided into four types, including oxidative fiber (I and IIa), intermediate fiber (IIx), and glycolytic fiber (IIb) [43]. Skeletal muscle rich in oxidative fiber has been reported to have a red appearance, higher myoglobin content, and better taste and flavor [44]. In the present study, results showed that CGA-supplemented diet promoted the mRNA levels of MyHC IIa and MyHC IIx, whereas it reduced the mRNA level of MyHC IIb. This would imply that the inclusion of dietary CGA in diet did promote a shift in muscle fiber type from glycolytic fiber to oxidative fiber. In view of the foregoing, CGA has the potential to serve as an effective functional feed additive to improve meat quality.

The advantage of pork as a food product is that it can provide the human body with high levels of nutrient-rich glycogen, fat, and protein [45]. Previous studies have demonstrated that CGA could decrease hepatocellular glucose output, whereas it enhances glucose uptake in skeletal muscle through the activation of Ca^2+^/calmodulin-dependent protein kinase phosphorylation [46]. In one of the studies with humans, chlorogenic acid-rich coffee components have been shown to increase muscles glucose levels, glycogen resynthesis rates, and glycogen accumulation [47]. Consistent with previous studies, this study shows that supplemental 50 mg kg^−1^ dietary CGA resulted in a significant increased glycogen content in LT. Moreover, dietary 100 mg kg^−1^ CGA supplementation markedly increased the content of muscle crude fat, illustrating excess glucose taken up by skeletal muscle is converted into fatty acids and stored as fat. It has been well documented that intramuscular fat content directly determines physical factors including juiciness and tenderness of pork [48]. In addition, dietary CGA elevated the mRNA level of ACC, which may explain the increased muscle crude fat content in the present experiment. Protein plays a key role in body growth and development, and the amino acids are the main component of protein molecules. Importantly, the amino acid levels and composition are responsible for meat quality and flavor [49]. Recently, the addition of phytochemical extract rich in polyphenol to feed is considered to be an effective means to improve the amino acid composition of pork [50]. In this study, it was observed that CGA-supplemented diet increased the concentration of total amino acid. These results are similar to the findings of a previous study in grass carp [51]. Specifically, results showed that pigs fed 50 mg/kg CGA had increased concentration of His in LD muscle. As an essential amino acid for infants and children, His is essential for body development. Additionally, the FAA content in muscle can partially explain the flavor of meat [52]. The present study demonstrated that a CGA-supplemented diet markedly increased the FAA content in LT. This further suggests that dietary CGA may not only alter overall nutrient value, but also increase flavor substances to profitably affect pork quality.

To date, little is known about the effectiveness of feeding pigs with the CGA on meat flavor and no studies investigated the underlying mechanisms. It is well known that taste active components such as IMP, glutamic acid, and some umami peptides contribute to meat flavor. Moreover, as a flavor enhancer, IMP is approximately 50 times more effective than monosodium glutamate [53]. Thus, IMP is used as an essential quality index for evaluating meat flavor, on a global scale. In this study, although dietary CGA was not observed to increase the content of IMP in muscle as in previous studies [16,50], CGA decreased the content of bitter substances produced by IMP metabolism, such as inosine and hypoxanthine. According to the description of Terasaki et al., the degradation rate of IMP in the IMP → inosine → hypoxanthine pathway depends on the dephosphorylation of IMP by 5′-nucleotidase [54]. This study found for the first time that a CGA-supplemented diet decreased the mRNA level of 5′-nucleotidase (NT5C), regarded as being a key nucleotide metabolism enzyme responsible for the reaction IMP → Inosine, but increased the mRNA level of hypoxanthine phosphoribosyl transferase 1 (HPRT1). The higher enzyme activity of HPRT1 suggests an improved potential for reversible phosphorylation of intracellular hypoxanthine to IMP in muscle [55]. In this sense, it may be supposed that dietary CGA improved the meat flavor by coordinating the mechanism of the subsequent IMP degradation.

As mentioned above, the addition of dietary natural antioxidants such as apple polyphenol (APPs) in pig diet can improve pork quality by mediating antioxidative status [50], suggesting that an association exists between the antioxidant compounds derived directly and indirectly from the diet and improvement of meat quality. CGA, one of the essential ingredients of APPs, has been described as a promising antioxidant. The mechanism of chlorogenic acid is mainly due to the fact that its hydrogen atoms located on aromatic residues can combine with free radicals to form phenoxy, which is quickly stabilized by resonance stabilization [14]. Li et al., in a study in which pigs were fed 1000 mg kg^−1^ chlorogenic acid-enriched extract (CGAE), observed increased T-SOD and T-AOC activities and SOD1 mRNA level in LT, which suggest an increased antioxidative ability [15]. Similarly, the findings of this study demonstrated that dietary CGA supplementation in finishing pigs can beneficially decrease MDA concentration in muscle tissues, while increasing muscle GSH-Px content as well as serum GSH-Px, CAT, and T-SOD levels. According to the literature, coffee constituents enable Nrf2 to be translocated from cytoplasmic Keap1 to induce the expression of concomitantly antioxidant genes in the nucleus [56]. In order to support the view that CGA plays a regulatory role in the nuclear factor erythroid 2-related factor 2 (Nrf2) pathway, the mRNA expression levels of the antioxidant-related genes were determined and it was found dietary CGA supplementation increased mRNA levels of *NQO-1*, *HO-1*, and *Nrf2* in muscle, which is consistent with the findings of previous study in finishing pigs [16]. Thus, it was concluded that dietary CGA could elevate antioxidant capacity to improve pork quality, which may be related to the Nrf2 signaling pathway.

## 5. Conclusions

The present findings showed that dietary CGA has a beneficial influence on carcass quality parameters, and can enhance nutritional value and flavor of pork. Furthermore, dietary CGA likely improved meat quality by increasing muscle antioxidant capacity and inducing a muscle fiber-type transition toward more slow-twitch fibers. Additionally, it was also found that dietary CGA supplementation regulates the mRNA abundance of metabolism-related and antioxidant-related gene transcripts in LT, which provides a molecular basis for improved meat quality. These results will also lay the foundation for the application of dietary CGA in pig production.

## Figures and Tables

**Table 1:** Composition and nutrient levels of the experimental diets (air-dry basis, %).

Items	Phase 1: 25–50 kg	Phase 2: 50–75 kg	Phase 3: 75–100 kg	Phase 4: 100–135 kg
Ingredient (%)				
Corn, 7.8% CP	76.30	79.20	82.85	85.88
Soybean meal, 44.2% CP	16.40	14.50	12.00	9.25
Soybean oil	1.50	1.85	1.85	1.85
Fish meal, 68%	2.65	1.60	0.70	0.70
L-Lys HCl, 98.5%	0.44	0.45	0.35	0.26
DL-Methionine, 99%	0.07	0.09	0.05	0.01
L-Threonine, 98.5%	0.15	0.12	0.12	0.08
L-Tryptophan, 98%	0.03	0.06	0.03	0.02
Choline chloride, 50%	0.15	0.15	0.15	0.15
Calcium bicarbonate	0.70	0.45	0.40	0.35
Dicalcium phosphate	0.96	0.88	0.85	0.80
NaCl	0.30	0.30	0.30	0.30
Mineral premix ^1^	0.30	0.30	0.30	0.30
Vitamin premix ^2^	0.05	0.05	0.05	0.05
Total	100	100	100	100
Nutrient levels ^3^				
Digestible energy, Mcal/kg	3.35	3.39	3.40	3.41
Crude protein, %	15.71	14.11	12.57	11.48
Crude fat, %	4.63	5.00	5.05	5.11
Crude fibre, %	2.74	2.69	2.62	2.52
Calcium, %	0.92	0.64	0.55	0.49
Total phosphorus, %	0.55	0.50	0.46	0.44
Non-phytic acid phosphor, %	0.32	0.27	0.23	0.22
SID Lys, %	0.99	0.91	0.74	0.61
SID Met, %	0.30	0.30	0.24	0.19
SID Cys, %	0.18	0.17	0.16	0.15
SID Met+Cys, %	0.48	0.47	0.40	0.33
SID Thr, %	0.58	0.51	0.46	0.39
SID Trp, %	0.17	0.18	0.14	0.11

^1^ The mineral premix provided the following per kg of diet: Fe (FeSO_4_·H_2_O), 40 mg; Cu (CuSO_4_·5H_2_O), 3 mg; Zn (ZnSO_4_·H_2_O), 50 mg; Mn (MnSO_4_·H_2_O), 2 mg; I (KI), 0.14 mg; Se (Na_2_SeO_3_), 0.15 mg. ^2^ The vitamin premix provided the following per kg of diet: Vitamin A, 9000 IU; Vitamin D_3_, 3000 IU; Vitamin E, 20.0 IU; Vitamin K_3_, 3.0 mg; Vitamin B_1_, 1.5 mg; Vitamin B_2_, 4.0 mg; Vitamin B_6_, 3.0 mg; Vitamin B_12_, 0.02 mg; Niacin, 30 mg; Pantothenic acid, 15 mg; Folic acid, 0.75 mg; Biotin, 0.1 mg. ^3^ Nutrient levels were calculated values.

**Table 2:** Primers used for real-time PCR analysis.

Gene ^1^	Accession Number	Primer Sequences (5′-3′) ^2^	Size/bp	AT ^3^, °C
*MyHCI*	NM_213855.1	F: GTTTGCCAACTATGCTGGGG	95	57
		R: TGTGCAGAGCTGACACAGTC		
*MyHC IIa*	NM214136.1	F: CTCTGAGTTCAGCAGCCATGA	127	58
		R: GATGTCTTGGCATCAAAGGGC		
*MyHc IIb*	NM_001123141.1	F: GAGGTACATCTAGTGCCCTGC	83	56.5
		R: GCAGCCTCCCCAAAAATAGC		
*MyHC IIx*	NM_001104951.2	F: TTGACTGGGCTGCCATCAAT	111	57
		R: GCCTCAATGCGCTCCTTTTC		
*PEPCK*	NM_001123158	F: TCAGCACGACTCCAGCCTTCA	122	59
		R: GCTCAAGCAGTCTGGGCATTCT		
*FASN*	NM_001099930.1	F: CTACGAGGCCATTGTGGACG	146	58.5
		R: AGCCTATCATGCTGTAGCCC		
*ACC1*	XM_021066238.1	F: AGCAAGGTCGAGACCGAAAG	169	57
		R: TAAGACCACCGGCGGATAGA		
*NT5C2*	XM_001927095.4	F: ACCAGTACCAGGGCACCATCT	263	61.8
		R: TCCCACGACTTCATTACCAACAG		
*AMPD1*	XM_005655414.3	F: AGGTCTTTATCGGGCATTGTG	266	58.9
		R: CTTTGCTGGCTGCTTCTTCAT		
*HPRT1*	NM_001032376.2	F: CCAGCGTCGTGATTAGTGATG	134	61
		R: AGCAAGCCGTTCAGTCCTGTC		
*HO-1*	NM_001004027.1	F: AGCACTCACAGCCCAACAGCA	164	64.2
		R: GTGGTACAAGGACGCCATCACC		
*NQO1*	NM_001159613.1	F: GGAAGAAACGCCTGGAGAATA	171	58.2
		R: GGATGGATTTGCCCAAGTGAT		
*KEAP1*	XM_005654811.3	F: ACAAACCGCCTCAACTCAGCA	281	61.6
		R: CCATCATAGCCTCCGAGAACATAG		
*NRF2*	XM_021075133.1	F: TCCACAGAAGACTCAAGCCAGAT	173	61
		R: GGCCCATACAGAAGTTCAGACAG		
*β* *-Actin*	XM_003124280.5	F: TGGAACGGTGAAGGTGACAGC	177	61
		R: GCTTTTGGGAAGGCAGGGACT		

^1^ *MyHC I* = Myosin heavy chain I, *MyHC IIa* = Myosin heavy chain IIa, *MyHC IIb* = Myosin heavy chain IIb, *MyHC IIx* = Myosin heavy chain IIx, *PEPCK* = Phosphoenolpyruvate carboxykinase, *FASN* = Fatty acid synthase, *ACC1* = Acetyl CoA carboxylase 1, *NT5C2* = 5′-nucleotidase, cytosolic II, *AMPD1* = adenosine monophosphate deaminase 1, *HPRT1* = hypoxanthine phosphoribosyltransferase 1, *HO-1* = heme oxygenase 1, *NQO1* = NAD(P)H quinone dehydrogenase 1, *KEAP1* = kelch-like ECH-associated protein 1, *NRF2* = NFE2-like bZIP transcription factor 2, *β-Actin* = beta actin. ^2^ F = forward primer; R = reverse primer; ^3^ AT = annealing temperature.

**Table 3:** Effect of dietary CGA supplementation on the growth performance of finishing pigs.

Item ^1^	CGA (mg/kg)				SEM	*p*-Value
	0	25	50	100		
Live weight, kg						
0 d	26.43	26.81	26.42	26.79	0.37	0.96
29 d	47.65	48.60	48.10	49.55	0.67	0.85
53 d	68.23	72.11	71.30	71.85	0.98	0.88
80 d	95.05	99.43	99.40	101.40	1.39	0.08
100 d	122.19 ^b^	123.83 ^ab^	121.00 ^b^	127.10 ^a^	1.70	0.04
ADG, g/d						
0 d–29 d	731.90	751.55	748.28	783.45	21.02	0.86
29 d–53 d	912.71	873.13	966.67	929.17	37.95	0.79
53 d–80 d	894.26	1022.04	1040.74	1094.44	61.71	0.81
80 d–100 d	1117.50	1055.00	1120.00	1285.00	62.28	0.49
0 d–100 d	943.25	990.45	956.00	1002.70	16.76	0.10
ADFI, g/d						
0 d–29 d	1560.31	1611.1	1687.87	1656.60	23.07	0.23
29 d–53 d	2073.39	1949.54	2180.52	2195.64	35.61	0.08
53 d–80 d	2786.47	2882.22	2992.70	3080.43	53.52	0.24
80 d–100 d	3305.80	3853.30	3435.05	3560.05	136.62	0.57
0 d–100 d	2363.91	2483.96	2507.85	2564.23	34.54	0.95
FCR, kg/kg						
0 d–29 d	2.13	2.14	2.26	2.12	0.08	0.64
29 d–53 d	2.30	2.34	2.27	2.37	0.10	0.57
53 d–80 d	2.89	2.55	2.90	2.82	0.34	0.71
80 d–100 d	2.67	3.27	3.08	2.93	0.44	0.29
0 d–100 d	2.50	2.51	2.63	2.57	0.04	0.47

^a, b^ Within a row, values with different superscript letters differ (*p* < 0.05). ^1^ Data are means of 10 replicates per treatment. ADG = average daily gain; ADFI = average daily feed intake; FCR = feed conversion ratio.

**Table 4:** Effect of dietary CGA supplementation on nutrient digestibility of finishing pigs.

Item	Chlorogenic Acid Inclusion Level (mg/kg)				SEM	*p*-Value
	0	25	50	100		
Energy	85.48	83.32	85.67	83.99	0.43	0.08
Crude protein	82.18	79.75	83.13	80.44	0.45	0.58
Ether extract	70.43	66.68	71.04	70.02	1.33	0.26
Dry matter	91.28 ^a^	90.49 ^ab^	91.48 ^a^	90.00 ^b^	0.20	0.05
Ash	62.27	61.73	67.28	67.85	0.98	0.55

^a, b^ Within a row, values with different superscript letters differ (*p* < 0.05).

**Table 5:** Effect of dietary CGA supplementation on serum biochemical indexes of finishing pigs.

Item ^1^	Chlorogenic Acid Inclusion Level (mg/kg)				SEM	*p*-Value
	0	25	50	100		
LDL-C, mmol/L	0.80	0.73	1.05	0.96	0.06	0.15
HDL-C, mmol/L	0.22	0.20	0.26	0.25	0.02	0.56
GLU, mmol/L	4.59	4.88	4.59	4.56	0.22	0.96
T-CHO, mmol/L	1.70	1.62	1.92	1.89	0.07	0.34
TG, mmol/L	0.45	0.46	0.55	0.49	0.03	0.61

^1^ Data are means of 5 replicates per treatment. LDL-C = low-density lipoprotein cholesterol; HDL-C = high-density lipoprotein cholesterol; GLU = glucose; T-CHO = total cholesterol; TG = triglyceride.

**Table 6:** Effect of dietary CGA supplementation on the carcass traits of finishing pigs.

Item ^1^	CGA (mg/kg)				SEM	*p*-Value
	0	25	50	100		
Carcass weight, kg	76.72	78.16	79.56	82.40	1.43	0.57
Carcass length, cm	104.30	105.20	106.60	107.30	0.91	0.68
Dressing percentage, %	62.80 ^b^	65.20 ^ab^	67.20 ^a^	65.00 ^ab^	0.75	0.05
Eye muscle area, cm^2^	66.31	63.45	62.31	68.52	1.60	0.54
Average backfat depth, mm	29.64	31.80	31.65	33.66	1.67	0.89
Carcass lean percentage, %	58.85	58.60	59.78	58.29	0.54	0.80
Skin weight, kg	7.38	7.07	6.75	7.88	0.40	0.78
Perirenal fat, kg	0.94 ^b^	1.14 ^ab^	1.37 ^a^	1.37 ^a^	0.06	0.03

^a, b^ Within a row, values with different superscript letters differ (*p* < 0.05). ^1^ Data are means of 5 replicates per treatment.

**Table 7:** Effect of dietary CGA supplementation on the physical traits of *longissimus thoracis* in finishing pigs.

Item ^1^	Chlorogenic Acid Inclusion Level (mg/kg)				SEM	*p*-Value
	0	25	50	100		
pH_45min_	6.24	6.15	6.29	6.14	0.64	0.83
pH_24h_	5.50 ^b^	5.51 ^b^	5.62 ^a^	5.51 ^b^	0.02	0.01
L*_45min_	43.32	43.27	44.43	44.11	0.38	0.66
a*_45min_	8.02	8.51	8.26	7.80	0.24	0.87
b*_45min_	6.42	6.45	6.70	6.51	0.12	0.85
L*_24h_	57.90	57.53	54.33	56.62	0.79	0.41
a*_24h_	11.04	10.86	10.12	10.10	0.18	0.13
b*_24h_	9.41	9.70	8.72	8.57	0.23	0.25
Drip loss, %	2.01	1.98	1.93	1.99	0.03	0.82
Cooking loss, %	35.66	33.80	34.54	35.10	0.59	0.75
Shear force, N	10.37	9.13	9.22	8.91	0.28	0.20

^a, b^ Within a row, values with different superscript letters differ (*p* < 0.05). ^1^ Data are means of 5 replicates per treatment. L* = lightness; a* = redness; b* = yellowness.

**Table 8:** Effect of dietary GCA supplementation on *longissimus thoracis* muscle chemical composition of finishing pigs.

Item ^1^	Chlorogenic Acid Inclusion Level (mg/kg)				SEM	*p*-Value
	0	25	50	100		
Muscle glycogen content, %	1.03 ^b^	1.32 ^ab^	1.49 ^a^	1.26 ^ab^	0.06	0.05
Muscle crude fat content, %	1.61 ^b^	2.59 ^ab^	2.64 ^ab^	3.03 ^a^	0.19	0.02
Muscle crude protein content, %	23.99	23.96	23.36	23.91	0.18	0.57
Inosine-5′-monophosphate, nmol/L	0.44	0.45	0.46	0.47	0.01	0.16
Hypoxanthine, pmol/L	162.61 ^a^	119.60 ^ab^	113.92 ^ab^	102.68 ^b^	20.17	0.04
Inosine, pmol/L	57.48 ^a^	57.13 ^a^	51.64 ^b^	48.97 ^b^	2.57	<0.01

^a, b^ Within a row, values with different superscript letters differ (*p* < 0.05). ^1^ Data are means of 5 replicates per treatment.

**Table 9:** Effect of dietary GCA supplementation on amino acid composition of *longissimus thoracis* muscle.

Item ^1^	Chlorogenic Acid Inclusion Level (mg/kg)				SEM	*p*-Value
	0	25	50	100		
Lys	7.15	7.16	7.09	7.16	0.07	0.98
Phe	3.09	3.11	3.06	3.09	0.03	0.96
Met	2.20	2.20	2.15	2.19	0.03	0.95
Thr	3.61	3.86	3.85	3.75	0.04	0.33
Val	3.96	3.99	3.97	4.07	0.05	0.87
Leu	6.49	6.46	6.40	6.47	0.07	0.98
Ile	3.72	3.70	3.66	3.71	0.04	0.96
Arg	4.79	4.76	4.69	4.71	0.05	0.90
His	3.32 ^b^	3.57 ^ab^	3.68 ^a^	3.58 ^ab^	0.05	<0.01
EAA	38.48	38.67	38.37	38.55	0.38	0.99
Asp	7.45	7.91	7.82	7.92	0.07	0.21
Glu	12.66	12.58	12.84	12.97	0.13	0.40
Gly	3.42	3.58	3.54	3.62	0.04	0.07
Ala	4.12	4.33	4.23	4.32	0.04	0.23
Pro	3.24	3.35	3.46	3.48	0.05	0.13
Ser	3.17	3.29	3.23	3.25	0.04	0.54
Tyr	2.71	2.69	2.68	2.70	0.03	0.99
Cys	0.38	0.31	0.36	0.41	0.02	0.58
TAA	73.11 ^b^	78.18 ^ab^	77.69 ^ab^	78.77 ^a^	0.69	0.05
FAA	36.85 ^b^	38.91 ^ab^	38.97 ^ab^	39.31 ^a^	0.36	0.02

^a, b^ Within a row, values with different superscript letters differ (*p* < 0.05). ^1^ Data are means of 5 replicates per treatment. EAA, essential amino acids = Lys + Met + Thr + Val + Leu + Ile + Tyr + Phe + His + Arg; NEAA, nonessential amino acids = Arg + His + Asp + Glu + Ala + Pro + Ser + Cys; FAA, flavored amino acids = Glu + Asp + Ala + Ser + Thr + Pro + Gly.

**Table 10:** Effect of dietary CGA supplementation on antioxidant capacity of finishing pigs.

Item ^1^	Chlorogenic Acid Inclusion Level (mg/kg)				SEM	*p*-Value
	0	25	50	100		
Serum						
MDA, nmol/mL	2.33	2.21	2.25	1.86	0.08	0.19
T-AOC, U/mL	1.20	1.18	1.80	2.21	0.18	0.14
CAT, U/mL	17.20 ^c^	18.96 ^bc^	25.95 ^ab^	30.10 ^a^	1.61	<0.01
GSH-Px, U/mL	71.91 ^c^	78.55 ^bc^	99.65 ^a^	92.61 ^ab^	3.66	<0.01
T-SOD, U/mL	70.07 ^c^	74.42 ^abc^	75.49 ^ab^	78.61 ^a^	1.09	0.03
Muscle						
MDA, nmol/mL	0.08 ^a^	0.07 ^ab^	0.04 ^c^	0.06 ^b^	0.01	0.05
T-AOC, U/mL	0.25	0.31	0.27	0.26	0.02	0.61
CAT, U/mL	8.60	9.37	8.71	9.99	0.33	0.44
GSH-Px, U/mL	107.68 ^b^	117.18 ^b^	130.43 ^b^	245.29 ^a^	14.86	<0.01
T-SOD, U/mL	102.14	110.91	103.95	106.65	7.40	0.98

^a, b, c^ Within a row, values with different superscript letters differ (*p* < 0.05). ^1^ Data are means of 5 replicates per treatment. MDA = malondialdehyde; CAT = catalase; T-AOC = total antioxidant capacity; T-SOD = total superoxide dismutase; GSH-Px = glutathione peroxidase.

## Data Availability

Data is contained within the article and available upon request from the corresponding author.
